# Artificial Intelligence-Assisted CBCT Evaluation of Maxillary Sinus, Nasal Cavity, and Nasolacrimal Duct Volumes in Unilateral and Bilateral Cleft Lip and Palate Patients

**DOI:** 10.3390/jcm15176680

**Published:** 2026-08-28

**Authors:** Anıl Demirel, Fırat Oğuz, Handan Göze Oğuz, Sabahattin Bor, Hamzahan Solak

**Affiliations:** 1 Department of Orthodontics, Faculty of Dentistry, İnönü University, Malatya 44280, Türkiye; a.demirel6060@hotmail.com (A.D.); venaroshan@gmail.com (S.B.); 2 Private Clinic, Malatya 44280, Türkiye; aydin_handan_01@hotmail.com; 3 Department of Oral and Maxillofacial Radiology, Faculty of Dentistry, İnönü University, Malatya 44280, Türkiye; hamzahan.solak@inonu.edu.tr

**Keywords:** cleft lip and palate, CBCT, artificial intelligence, maxillary sinus, nasal cavity, nasolacrimal duct, orthodontics

## Abstract

**Background/Objectives**: This study aimed to evaluate the volumes of the maxillary sinus, nasal cavity, and nasolacrimal duct in individuals with unilateral and bilateral cleft lip and palate (CLP) using an artificial intelligence-assisted segmentation method and to compare the findings with those of healthy controls. **Methods**: This retrospective study included 25 unilateral CLP patients, 20 bilateral CLP patients, and 25 healthy controls. Maxillary sinus, nasal cavity, and nasolacrimal duct volumes were measured on cone-beam computed tomography (CBCT) images using an artificial intelligence-assisted segmentation method. Total volume differences among groups were analyzed using analysis of covariance (ANCOVA) adjusted for age and sex. Side-based comparisons were performed using linear mixed-effects models. Benjamini–Hochberg correction was applied for multiple comparisons. **Results**: The control group exhibited greater maxillary sinus, nasal cavity, and nasolacrimal duct volumes than the CLP groups. ANCOVA demonstrated nominally significant group differences for the maxillary sinus (*p* = 0.008), nasal cavity (*p* = 0.034), and nasolacrimal duct (*p* = 0.023); however, these differences were no longer significant after Benjamini–Hochberg correction (q > 0.05). Pairwise comparisons showed greater maxillary sinus volumes in controls than in the unilateral (*p* = 0.037) and bilateral CLP groups (*p* = 0.007), and greater nasal cavity volumes than in the unilateral (*p* = 0.049) and bilateral CLP groups (*p* = 0.045). Nasolacrimal duct volume differed only between the bilateral CLP and control groups (*p* = 0.021). No significant volumetric differences were found between unilateral and bilateral CLP groups. **Conclusions**: Although individuals with CLP showed a trend toward reduced maxillary sinus, nasal cavity, and nasolacrimal duct volumes, the total-volume differences did not remain significant after correction for multiple comparisons; reductions relative to the control sides persisted after correction only for the maxillary sinus and the nasolacrimal duct in the side-based analysis. No significant differences were observed between unilateral and bilateral CLP or between the two sides within any group.

## 1. Introduction

Cleft lip and palate (CLP) is a congenital craniofacial anomaly that results from the failure of fusion between the maxillary and medial nasal processes during embryonic development, particularly during the formation of the primary palate [1,2]. Cleft lip, resulting from defective fusion of the frontonasal and maxillary processes, affects the lip, alveolus, and nasal floor to varying degrees, whereas cleft palate arises from incomplete fusion of the palatal shelves and presents as a defect involving the hard palate, soft palate, or both [3]. These anomalies may occur unilaterally or bilaterally and may involve different combinations of the alveolus and palate [4]. The etiology of CLP is multifactorial, resulting from the interaction between genetic predisposition and environmental factors. Poor maternal nutrition, hormonal disorders, medications, toxic agents, and various biological factors are among the major environmental risk factors [5].

Patients with CLP exhibit characteristic anatomical and morphological alterations associated with various functional impairments. Facial deformities, feeding difficulties, dental anomalies, malocclusion, and speech and hearing disorders have all been reported in these patients [6]. These findings indicate that CLP is not merely an aesthetic condition but rather a complex craniofacial anomaly associated with multiple functional impairments [7,8].

Various nasal deformities, including maxillary sinus anomalies, nasal septal deviation, turbinate hypertrophy, narrowing of the nasal cavity, and airway obstruction, have been reported in patients with CLP [9,10]. Craniofacial clefts may also affect the anatomy of the nasolacrimal system [11]. Owing to the close anatomical relationship between the nasolacrimal duct and the lateral nasal wall, infections and various pathological conditions may result in obstruction of the nasolacrimal system [12,13]. Furthermore, CLP has been associated with a variety of abnormalities involving the paranasal sinuses [10], and morphological alterations of the maxillary sinus have been suggested to increase susceptibility to rhinological disorders, particularly sinusitis [2,14]. Therefore, evaluation of these anatomical alterations is of considerable importance during combined orthodontic and maxillofacial surgical treatment planning.

Cone-beam computed tomography (CBCT) is widely used for the evaluation of patients with CLP because it provides three-dimensional imaging with a relatively low radiation dose [2,15]. Although several studies have investigated maxillary sinus volume in individuals with CLP, no study has simultaneously evaluated the volumes of the maxillary sinus, nasal cavity, and nasolacrimal duct by comparing unilateral and bilateral CLP patients with healthy controls. Therefore, the present study aimed to compare the volumes of the maxillary sinus, nasal cavity, and nasolacrimal duct between patients with unilateral and bilateral CLP and healthy controls using CBCT, and to determine whether these structures represent meaningful indicators for assessing CLP-related morphological alterations. Accordingly, the following null hypotheses were tested:

**H_01_.**  *There are no significant differences in the total volumes of the maxillary sinus, nasal cavity, and nasolacrimal duct among the unilateral CLP, bilateral CLP, and control groups.*

**H_02_.**  *There are no significant differences in the volumes of the maxillary sinus, nasal cavity, and nasolacrimal duct between the cleft and non-cleft sides in the unilateral CLP group, or between the right and left sides in the bilateral CLP and control groups.*

**H_03_.**  *The volumes of the maxillary sinus, nasal cavity, and nasolacrimal duct between the cleft and non-cleft sides in the unilateral CLP group, and between the right and left sides in the bilateral CLP and control groups, are equivalent within the predefined ±10% equivalence margin.*

## 2. Materials and Methods

This study was approved by the Ethics Committee of İnönü University (Approval No. 2026/9759; Date: 7 April 2026). The study material consisted of a retrospective review of patient records from individuals who presented to the Department of Orthodontics, Faculty of Dentistry, İnönü University, for orthodontic treatment between 2021 and 2026. A total of 3728 patient records were retrospectively screened, among which 140 patients with unilateral or bilateral cleft lip and palate (CLP) were identified.

Only individuals with cone-beam computed tomography (CBCT) images of sufficient image quality and free of artifacts within the anatomical region of interest were included. Patients with a history of trauma or surgery involving the head and neck region, other than primary cleft repair procedures, infectious, allergic, or other pathological conditions affecting the paranasal sinuses, maxillary sinus fractures, or sinus pathology were excluded. In addition, individuals who had previously undergone orthodontic treatment, had maxillary sinus tumors, or presented with craniofacial deformities other than CLP were not included in the study. All patients had undergone primary cheiloplasty and palatoplasty. None had undergone secondary rhinoplasty or secondary maxillary surgery. Because alveolar bone grafting is a routine component of cleft care and was performed in many patients before referral, it was not used as an exclusion criterion.

Sample size calculation was performed using G*Power software (version 3.1; Heinrich Heine University Düsseldorf, Düsseldorf, Germany). Assuming an effect size of f = 0.40, a significance level of α = 0.05, and a statistical power of 80%, the minimum required sample size was calculated to be 66 participants [16]. A total of 70 individuals meeting the inclusion and exclusion criteria were ultimately enrolled. Although equal group sizes were initially planned, the lower availability of bilateral CLP cases resulted in a final sample comprising 25 unilateral CLP patients (17 left-sided and 8 right-sided clefts), 20 bilateral CLP patients, and 25 healthy controls.

CBCT images of all participants were acquired using the same imaging system (NewTom 5G, Verona, Italy). All tomographic datasets were exported in Digital Imaging and Communications in Medicine (DICOM) format. Individual patient files were then created, and the CBCT images were imported into the open-source ITK-SNAP software (version 4.4.0) for further analysis. Prior to volumetric assessment, head orientation was standardized for all images to ensure consistent evaluation. Volumetric analyses were performed using the nnInteractive deep learning–based interactive segmentation tool integrated into ITK-SNAP. The workflow was AI-assisted and operator-guided rather than fully automatic. The operator provided initial inputs to define the target anatomical structure, after which nnInteractive generated the segmentation. The resulting segmentation was reviewed in the axial, sagittal, and coronal planes. When incomplete segmentation or inaccurate boundaries were identified, additional operator-defined inputs were provided and the AI-assisted segmentation was reapplied. Manual interaction was limited to local corrections of anatomical boundaries when necessary rather than re-segmentation of the entire structure. Manual refinement was required mainly in narrow or poorly demarcated regions of the nasal cavity and was less frequently required for the maxillary sinus and nasolacrimal duct. Thus, AI primarily facilitated and accelerated segmentation, while the operator retained control over the final anatomical boundaries.

**Figure 1:**
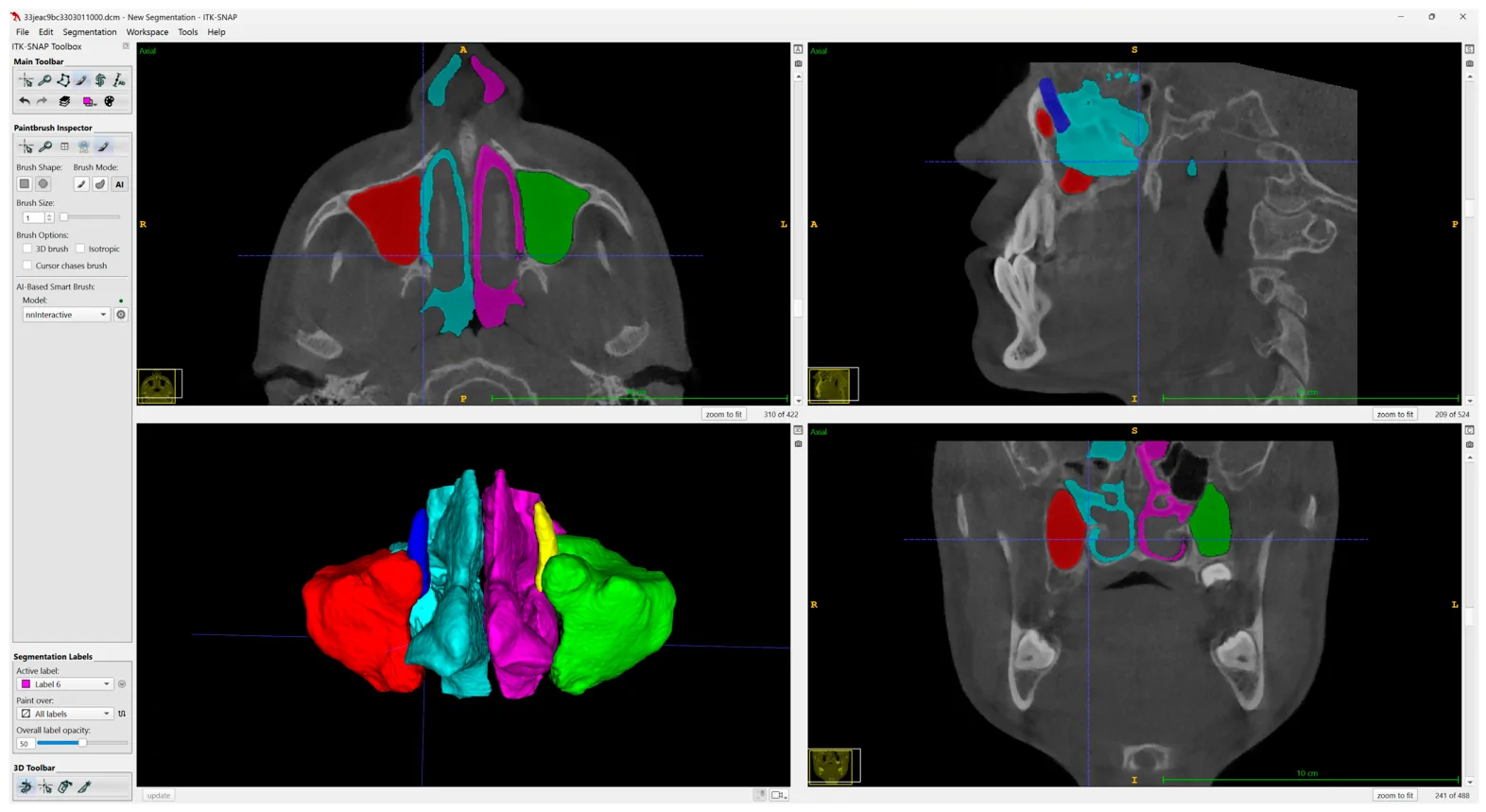
AI-assisted segmentation of the bilateral maxillary sinuses, nasal cavities, and nasolacrimal duct in ITK-SNAP. Representative axial, sagittal, coronal, and 3D views are presented.

For each patient, the right and left maxillary sinuses, nasal cavities, and nasolacrimal ducts were segmented separately. During maxillary sinus segmentation, the anatomical communications between the nasal cavity and the maxillary sinus were carefully examined in the axial, sagittal, and coronal planes to ensure accurate separation, and only the maxillary sinus cavity was included in the segmentation. Segmentation of the nasal cavity and nasolacrimal duct was performed according to the same principles, using their respective anatomical boundaries as references. Consequently, six individual segmentations (right and left maxillary sinus, nasal cavity, and nasolacrimal duct) were obtained for each participant (Figure 1). All primary segmentation procedures and volumetric measurements were performed by a single investigator (A.D.). For reliability assessment, a second investigator (F.O.) independently segmented the same subsample, blinded to the initial measurements.

**Figure 2:**
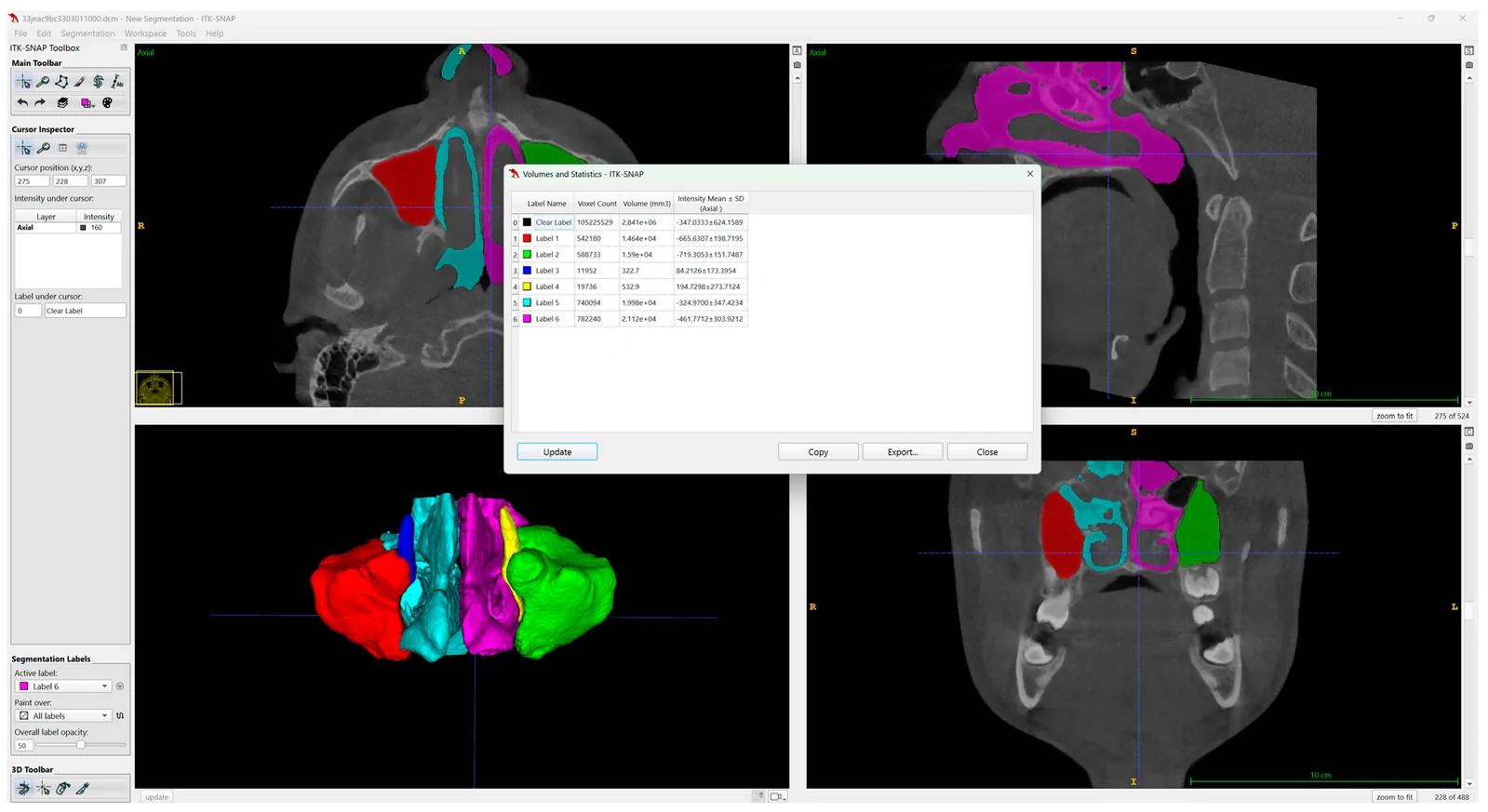
Three-dimensional reconstruction and volumetric analysis of the segmented bilateral maxillary sinuses, nasal cavities, and nasolacrimal duct using the Volumes and Statistics module in ITK-SNAP.

Upon completion of the segmentation process, three-dimensional reconstructions of each anatomical structure were generated, and volumetric measurements were automatically calculated using the Volumes and Statistics module of the ITK-SNAP software (Figure 2). Measurement reliability was assessed on the CBCT images of seven randomly selected participants: intra-observer reliability was based on repeat measurements performed by the same investigator (A.D.) after a one-month interval, and inter-observer reliability on independent measurements performed by a second investigator (F.O.). The obtained volumetric measurements were initially recorded in cubic millimeters (mm^3^) in Microsoft Excel (Microsoft Corp., Redmond, WA, USA), subsequently converted to cubic centimeters (cm^3^), and used for statistical analyses.

### Statistical Analysis

Statistical analyses were performed using R (version 4.5.1, R Foundation for Statistical Computing, Vienna, Austria) and RStudio (version 2026.6.0.242, Posit Software, PBC, Boston, MA, USA). Appropriate data transformations were applied when necessary based on the distributional characteristics of the volumetric data. Group comparisons were performed using analysis of covariance (ANCOVA) adjusted for age and sex. Bilateral measurements were analyzed using linear mixed-effects models with participant included as a random effect. Equivalence was assessed using the two one-sided tests (TOST) procedure. The Benjamini–Hochberg method was applied to adjust for multiple comparisons. Intra- and inter-observer reliability were evaluated using the intraclass correlation coefficient (ICC(2,1)), Dahlberg’s error, the standard error of measurement (SEM), the minimal detectable change at the 95% confidence level (MDC95), and Bland–Altman analysis. Statistical significance was set at *p* < 0.05. A sensitivity power analysis was also performed based on the final sample size to determine the minimum detectable effect size at 80% power and α = 0.05. Pairwise comparisons among the six subgroups were obtained as contrasts of estimated marginal means from the mixed-effects model, with degrees of freedom by the Kenward-Roger method and back-transformed to geometric-mean ratios with 95% confidence intervals; the Benjamini–Hochberg correction was applied within each anatomical structure.

## 3. Results

A total of 70 participants were included in the study, comprising 25 individuals with unilateral CLP, 20 with bilateral CLP, and 25 healthy controls. The demographic characteristics of the study groups are presented in Table 1. Significant differences were observed among the groups with respect to age and sex distribution (*p* < 0.001 and *p* = 0.001, respectively). Therefore, all group comparisons were adjusted for age and sex.

**Figure 3:**
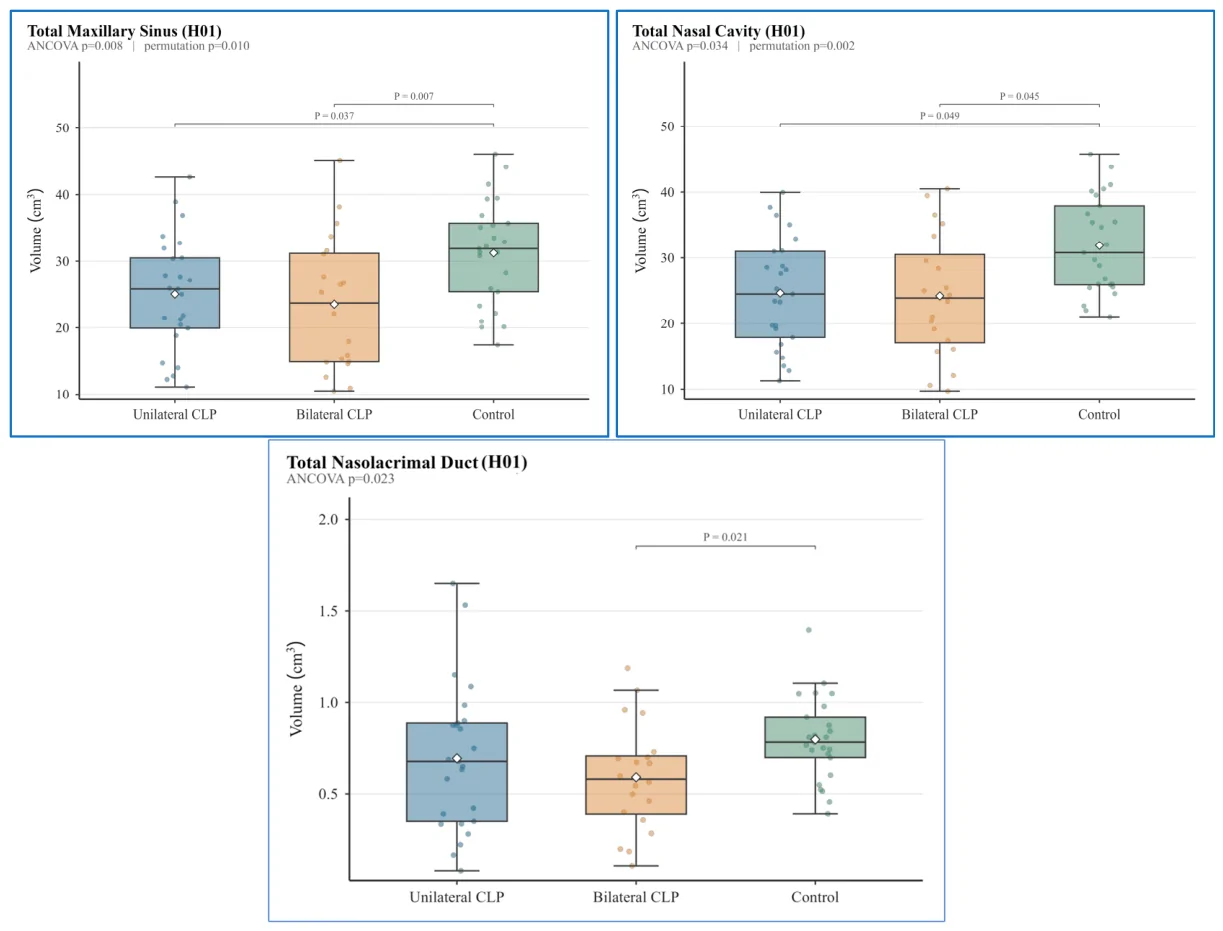
Boxplots showing the total volumes of the maxillary sinus, nasal cavity, and nasolacrimal duct in the unilateral CLP, bilateral CLP, and control groups.

When descriptive statistics of the total (right + left) volumes were examined, the total maxillary sinus volume was 25.01 ± 8.58 cm^3^, 23.53 ± 10.08 cm^3^, and 31.24 ± 7.84 cm^3^ in the unilateral CLP, bilateral CLP, and control groups, respectively. The corresponding total nasal cavity volumes were 24.58 ± 8.34 cm^3^, 24.15 ± 9.40 cm^3^, and 31.90 ± 7.35 cm^3^, whereas the total nasolacrimal duct volumes were 0.694 ± 0.40 cm^3^, 0.591 ± 0.30 cm^3^, and 0.798 ± 0.23 cm^3^, respectively (Figure 3).

Analysis of covariance (ANCOVA), adjusted for age and sex, demonstrated nominally significant group differences in maxillary sinus (*p* = 0.008, ηp^2^ = 0.15), nasal cavity (*p* = 0.034, ηp^2^ = 0.17), and nasolacrimal canal (*p* = 0.023, ηp^2^ = 0.09) volumes. However, none of these differences remained statistically significant after Benjamini–Hochberg correction (q > 0.05). Pairwise comparisons (Figure 3) showed that the control group had significantly greater maxillary sinus volumes than both the unilateral CLP (*p* = 0.037) and bilateral CLP (*p* = 0.007) groups. Similarly, nasal cavity volume was significantly greater in the control group than in the unilateral CLP (*p* = 0.049) and bilateral CLP (*p* = 0.045) groups. Nasolacrimal duct volume differed only between the bilateral CLP and control groups (*p* = 0.021). No significant differences were observed between the unilateral and bilateral CLP groups for the total volumes of any of the three anatomical structures (*p* > 0.05).

**Figure 4:**
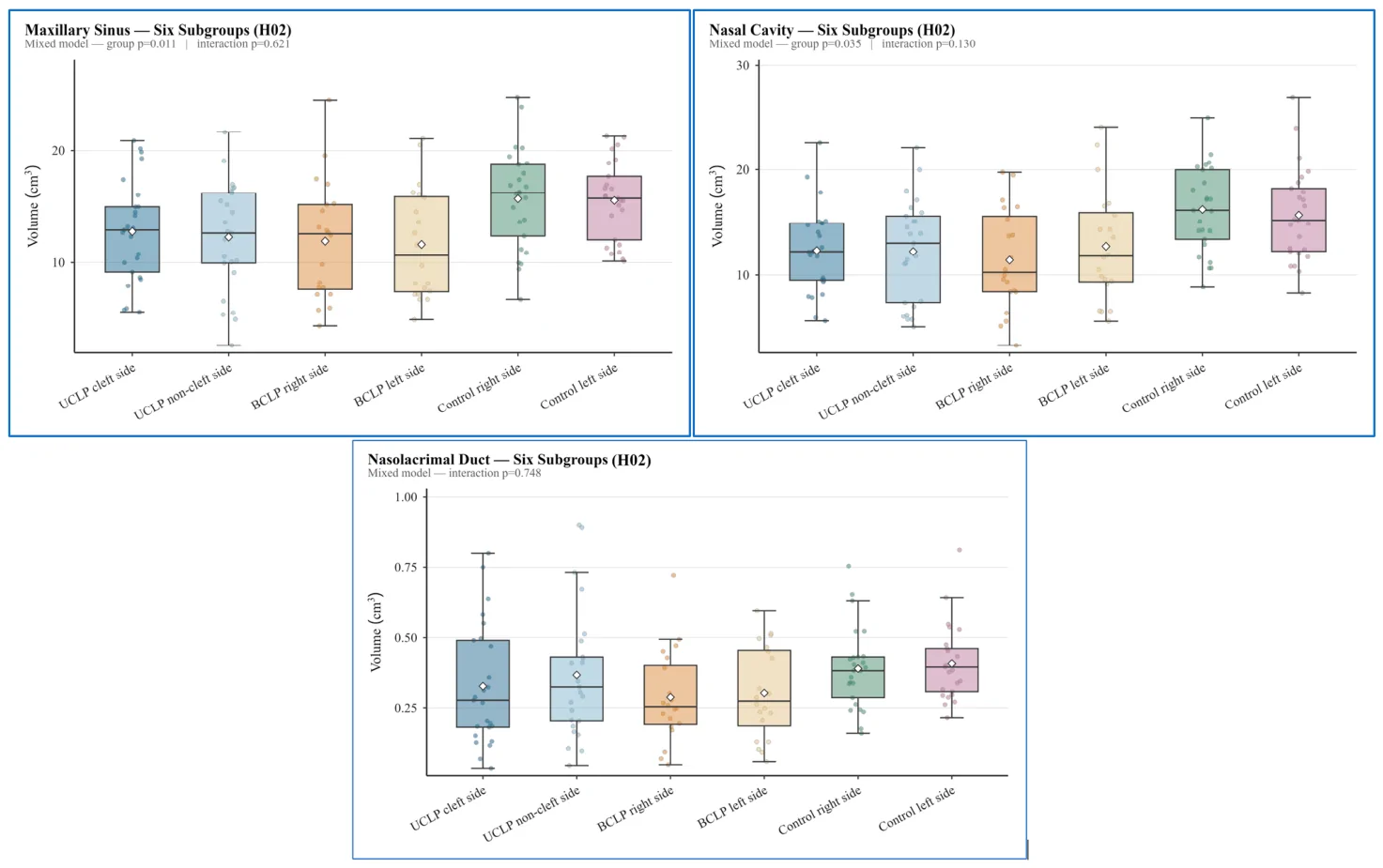
Boxplots showing side-specific volumes of the maxillary sinus, nasal cavity, and nasolacrimal duct across the six subgroups.

In the side-based analysis, maxillary sinus volumes in the unilateral CLP group were 12.75 ± 4.57 cm^3^ on the cleft side and 12.26 ± 4.83 cm^3^ on the non-cleft side. The corresponding nasal cavity volumes were 12.34 ± 4.09 cm^3^ and 12.24 ± 4.91 cm^3^, while nasolacrimal duct volumes were 0.327 ± 0.21 cm^3^ and 0.367 ± 0.23 cm^3^, respectively. In the bilateral CLP group, maxillary sinus volumes were 11.91 ± 5.34 cm^3^ on the right side and 11.62 ± 4.96 cm^3^ on the left side; nasal cavity volumes were 11.41 ± 4.87 cm^3^ and 12.74 ± 5.32 cm^3^; and nasolacrimal duct volumes were 0.288 ± 0.16 cm^3^ and 0.303 ± 0.16 cm^3^, respectively. In the control group, maxillary sinus volumes were 15.70 ± 4.51 cm^3^ on the right side and 15.55 ± 3.50 cm^3^ on the left side; nasal cavity volumes were 16.22 ± 4.07 cm^3^ and 15.68 ± 4.48 cm^3^; and nasolacrimal duct volumes were 0.390 ± 0.14 cm^3^ and 0.407 ± 0.13 cm^3^, respectively (Figure 4).

Linear mixed-effects model analysis adjusted for age and sex demonstrated a significant main effect of group for maxillary sinus (*p* = 0.011), nasal cavity (*p* = 0.035), and nasolacrimal duct (*p* = 0.022) volumes. However, the group × side interaction was not significant for any of the three anatomical structures (*p* = 0.621, *p* = 0.130, and *p* = 0.748, respectively) (Table 2).

Pairwise contrasts of estimated marginal means among the six subgroups showed that maxillary sinus volumes were significantly smaller in the unilateral CLP non-cleft side and in both bilateral CLP sides than in either control side (ratios 0.65–0.70; Benjamini–Hochberg-adjusted *p* = 0.023 for all six contrasts). For the nasolacrimal duct, volumes were significantly smaller in the unilateral CLP cleft side and in both bilateral CLP sides than in either control side (ratios 0.51–0.62; adjusted *p* = 0.038–0.049). For the nasal cavity, no contrast remained significant after correction (lowest adjusted *p* = 0.060). No significant differences were detected between the unilateral and bilateral CLP subgroups, or between the two sides within any group, for any of the three structures (Supplementary Table S1).

**Figure 5:**
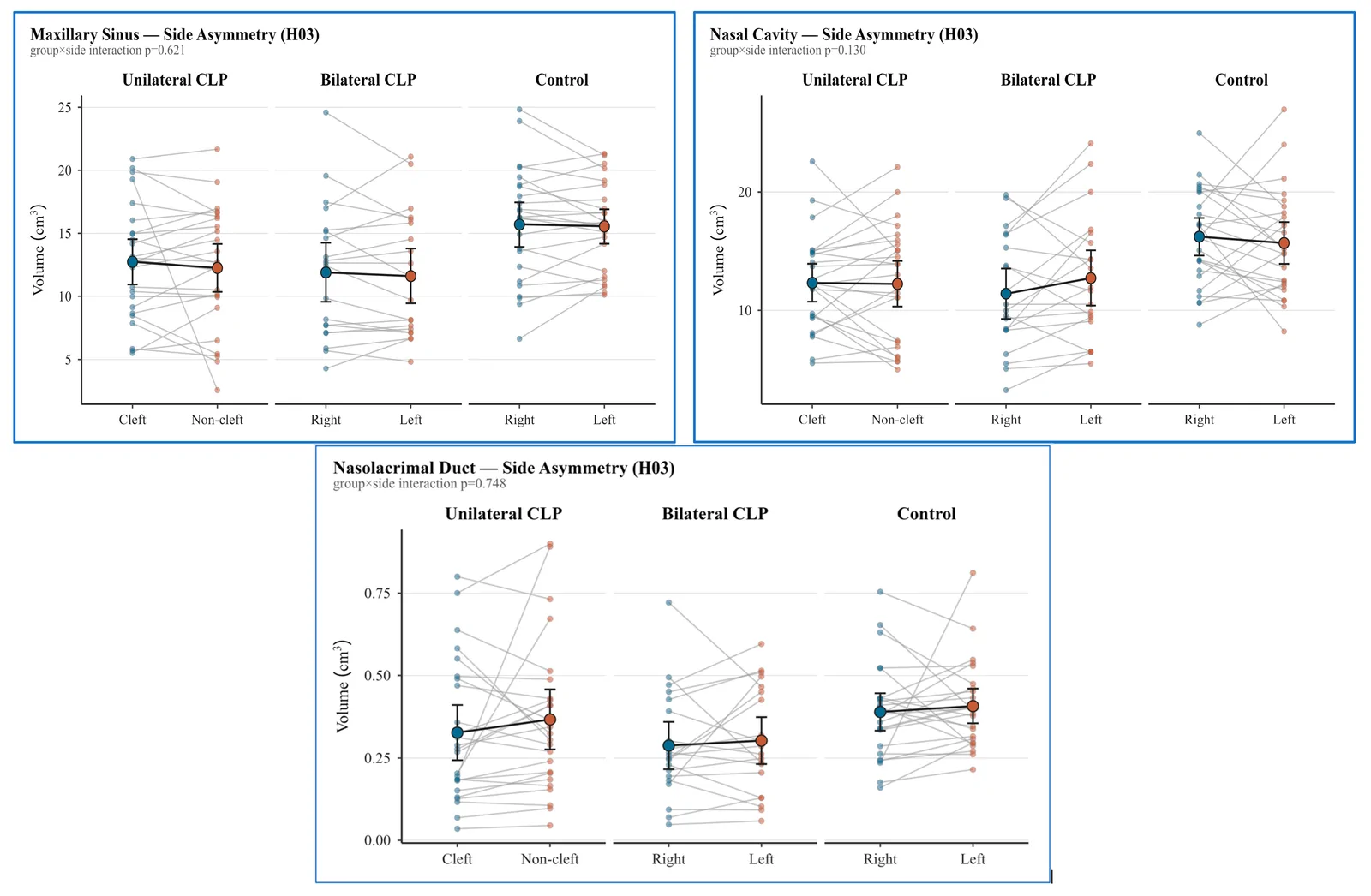
Paired comparisons of side-specific maxillary sinus, nasal cavity, and nasolacrimal duct volumes within the unilateral CLP, bilateral CLP, and control groups.

When side-based comparisons were performed within each group, no statistically significant asymmetry was observed between the cleft and non-cleft sides in the unilateral CLP group or between the right and left sides in the bilateral CLP and control groups with respect to maxillary sinus, nasal cavity, or nasolacrimal duct volumes (all Benjamini–Hochberg-adjusted *p* > 0.05). To distinguish the absence of a statistically significant difference from evidence of similarity between sides, equivalence was additionally assessed using the TOST procedure. As no established clinically meaningful equivalence threshold is available for these volumetric measurements, a ±10% margin was selected as a conservative proportional threshold for differences considered practically negligible. Based on this margin, equivalence was demonstrated only for maxillary sinus volumes in the bilateral CLP and control groups. No equivalence was established for nasal cavity or nasolacrimal duct volumes in any group. Cohen’s dz effect sizes were small for all comparisons (|dz| ≤ 0.34) (Table 3, Figure 5).

In the intra- and inter-observer reliability analyses, maxillary sinus volume measurements demonstrated excellent agreement in both the intra- and inter-observer assessments (ICC = 1.00). In contrast, reliability for the nasal cavity and nasolacrimal duct measurements ranged from moderate to excellent (ICC = 0.55–0.95), indicating greater variability in reproducibility. Dahlberg’s error and the standard error of measurement (SEM) were low, while Bland–Altman analyses showed that the vast majority of measurements were within the 95% limits of agreement, indicating the absence of systematic measurement bias.

## 4. Discussion

In the present study, the volumes of the nasal cavity, maxillary sinus, and nasolacrimal duct were evaluated three-dimensionally in individuals with unilateral and bilateral cleft lip and palate (CLP) and compared with those of healthy controls. Although the initial analyses revealed nominally significant differences among the groups with respect to the volumes of the investigated anatomical structures, these differences did not remain statistically significant after correction for multiple comparisons in the analysis of total volumes. Therefore, the first null hypothesis could not be rejected. In the side-based analysis, however, pairwise contrasts among the six subgroups remained significant after correction for the maxillary sinus and the nasolacrimal duct, with all significant contrasts occurring between a CLP subgroup and a control side. Furthermore, as no significant volumetric differences were observed between sides, the second null hypothesis was supported. However, because equivalence was demonstrated only in a limited number of comparisons, the third null hypothesis was only partially supported.

An important consideration when interpreting these findings is the significant age imbalance between the study groups. Although all between-group analyses were adjusted for age and sex, the control group was older than both CLP groups, and residual confounding related to developmental differences cannot be completely excluded. Because the maxillary sinus and nasal cavity continue to undergo growth and remodeling throughout adolescence, part of the observed volumetric differences may reflect age-related anatomical development rather than CLP alone. Therefore, the present findings should be interpreted with appropriate caution.

The development of the primary palate is closely associated with the formation of the medial portion of the maxilla, as well as the lip and nose. Therefore, structural alterations of the maxillary sinus may occur in individuals with CLP [17]. In addition, these individuals have been reported to be more susceptible to various rhinological disorders, particularly sinusitis, which may be associated with the presence of the cleft. Previous studies have also shown that patients with CLP exhibit reduced maxillary sinus height and depth, whereas nasal septal deviation, mucosal thickening, and maxillary sinus pathologies are more frequently observed [10,18]. Therefore, evaluation of maxillary sinus anatomy is of considerable clinical importance [14]. Historically, cephalometric radiographs [19,20] and computed tomography (CT) [8,21] have been used to evaluate the maxillary sinus. However, because cephalometric radiographs provide two-dimensional representations of three-dimensional anatomical structures, they have inherent limitations for volumetric assessment. Therefore, CBCT was used in the present study for volumetric evaluation.

In the present study, maxillary sinus volumes were greater in the control group than in both CLP groups. However, these differences did not remain statistically significant after correction for multiple comparisons. In addition, no significant volumetric differences were observed between the unilateral and bilateral CLP groups or between sides within any group. Equivalence between sides was demonstrated only in the bilateral CLP and control groups.

Previous studies have consistently reported smaller maxillary sinus volumes in individuals with CLP compared with healthy controls. Altun and Dumlu [18], reported significantly lower total maxillary sinus volumes in the CLP group than in the control group (21,232 mm^3^ vs. 23,763 mm^3^; *p* = 0.026). Similarly, Barbosa et al. [22], Yıldırım et al. [23], Demirtaş et al. [24], Erdur et al. [14], Tunç and Ünsal [25] and Altındağ et al. [26] also reported reduced maxillary sinus volumes in unilateral and/or bilateral CLP patients compared with controls. In the present study, maxillary sinus volumes were greater in the control group than in both CLP groups. However, these differences did not remain statistically significant after correction for multiple comparisons. In addition, no significant volumetric differences were observed between the unilateral and bilateral CLP groups.

With respect to comparisons between cleft types, the findings of Barbosa et al. [22] and Yıldırım et al. [23] who reported no significant volumetric differences between unilateral and bilateral CLP groups, are consistent with the results of the present study. In contrast, Altun and Dumlu [18] evaluated unilateral and bilateral CLP cases as a single group; therefore, the potential effect of cleft type on maxillary sinus volume could not be assessed.

Regarding side-based comparisons, the literature has reported inconsistent findings. Barbosa et al. [22], Erdur et al. [14] and Sayeste et al. [27], found no significant volumetric differences between the cleft and non-cleft sides in patients with unilateral CLP, which is consistent with our findings. In contrast, Yıldırım et al. [23], Eslami et al. [28], Rodrigues et al. [2] and Tunç and Ünsal [25], reported significantly smaller maxillary sinus volumes on the cleft side than on the non-cleft side. These discrepancies among studies may be attributed to differences in sample size and age distribution, the classification of unilateral and bilateral CLP cases, imaging and segmentation techniques, and the characteristics of the study populations. Taken together, the present findings do not provide statistically robust evidence for maxillary sinus volume differences according to cleft type or side.

One possible explanation for the discrepancies among studies may be differences in the segmentation methods employed. Kolodziejska et al. [29], compared manual and semi-automatic three-dimensional segmentation methods for measuring maxillary sinus volume in individuals with unilateral CLP and reported smaller maxillary sinus volumes in the CLP group. They also demonstrated that volumetric differences between manual and semi-automatic methods were more pronounced in the CLP group. The authors emphasized that semi-automatic three-dimensional segmentation may provide more reliable volumetric measurements in individuals with CLP because of the irregular morphology of the maxillary sinus. Similarly, the use of an artificial intelligence-assisted segmentation method in the present study may have contributed to more standardized and reproducible volumetric measurements in CLP cases with irregular anatomical boundaries.

From a clinical perspective, the relationship between maxillary sinus morphology and sinonasal disorders may also be relevant in patients with CLP. Although the pathogenesis of sinusitis in these patients remains unclear and is considered multifactorial, reduced maxillary sinus dimensions have been associated with chronic sinusitis in previous studies. Therefore, three-dimensional evaluation of maxillary sinus anatomy may provide complementary information for the rhinologic assessment of patients with CLP, particularly in the presence of recurrent or chronic sinonasal symptoms.

In the present study, nasolacrimal duct volume was lower in both CLP groups than in the control group, with the lowest volume observed in the bilateral CLP group. However, these differences did not remain statistically significant after correction for multiple comparisons. In addition, no significant volumetric differences were observed between the unilateral and bilateral CLP groups or between sides within any group.

The number of studies evaluating nasolacrimal duct morphology in individuals with CLP is limited. Most published studies have focused primarily on the linear dimensions of the duct, whereas volumetric assessments have been reported in only a few investigations. Furthermore, studies simultaneously evaluating both unilateral and bilateral CLP cases remain scarce. Consequently, the effects of CLP on nasolacrimal duct morphology have not yet been fully elucidated.

One of the first studies to investigate the three-dimensional volumetric characteristics of the nasolacrimal duct, Güler and Göksel [30], reported significantly smaller nasolacrimal duct volumes and transverse diameters on the cleft side than on the non-cleft side in patients with unilateral CLP. However, no significant side-to-side differences were observed in duct length, anteroposterior diameter, or duct angulation. Bozdemir and Yarbasi [31] compared unilateral and bilateral CLP cases with healthy controls and demonstrated significant differences in the transverse and anteroposterior diameters of the nasolacrimal duct, particularly in bilateral CLP cases. In addition, duct length on the affected side was significantly shorter in unilateral CLP patients than in controls. Similarly, Altun et al. [32], reported a narrower nasolacrimal duct diameter on the affected side in patients with unilateral CLP. In contrast, Duman Tepe et al. [33], compared the volumetric and morphological characteristics of the nasolacrimal duct between unilateral CLP patients and healthy controls and found no significant differences in duct volume, length, or transverse and anteroposterior diameters. The authors emphasized that these morphological variations may have clinical relevance during Le Fort osteotomies and other maxillofacial surgical procedures.

In the present study, pairwise comparisons demonstrated a significant difference in nasolacrimal duct volume only between the bilateral CLP and control groups (*p* = 0.021); however, this difference did not remain statistically significant after Benjamini–Hochberg correction. Moreover, no significant volumetric asymmetry was observed in the side-based analyses. In the side-based analysis, by contrast, the CLP subgroups differed significantly from the control sides after correction, whereas neither the two CLP subgroups nor the two sides within any group differed from each other. These findings suggest that the effects of CLP on the nasolacrimal duct may reflect generalized craniofacial developmental alterations rather than a localized side-specific effect. However, because the reliability of nasolacrimal duct measurements ranged from moderate to excellent, the present findings should be interpreted with appropriate caution, as measurement variability may have reduced the ability to detect subtle volumetric differences. Nevertheless, given the limited number of volumetric studies currently available and the variability in measurement methodologies, further studies with larger sample sizes and standardized three-dimensional volumetric assessment protocols are needed to validate these findings.

In the present study, nasal cavity volumes were lower in both CLP groups than in the control group. However, these differences did not remain statistically significant after correction for multiple comparisons. No significant volumetric differences were observed between the unilateral and bilateral CLP groups or between sides within any group.

Similar to the present study, Farzal et al. [34] reported smaller total nasal cavity volumes in both unilateral and bilateral CLP patients than in healthy controls. They also found no significant differences in total nasal cavity volume between unilateral and bilateral CLP groups and no significant volumetric asymmetry between the cleft and non-cleft sides in unilateral cases or between the right and left sides in bilateral cases [34].

In contrast, Inocentes et al. [35] reported that nasal cavity volume was significantly lower only in the unilateral CLP group compared with controls, whereas no significant differences were observed between the bilateral CLP and control groups or between the unilateral and bilateral CLP groups. Similarly, Sehrawat et al. [36] reported reduced nasal cavity volumes in patients with unilateral CLP compared with healthy controls. However, unlike the findings of the present study, they also demonstrated significantly smaller nasal cavity volumes on the cleft side than on the non-cleft side (*p* < 0.001). Although the findings for the unilateral CLP group are consistent with our results, the absence of reduced nasal cavity volume in the bilateral CLP group and the presence of significant side-to-side differences differ from our observations, in which lower nasal cavity volumes were identified in both CLP groups and no significant volumetric asymmetry was detected. These discrepancies may be explained by differences in sample characteristics, age and sex distribution, skeletal pattern, surgical protocols, and the anatomical boundaries used for nasal cavity segmentation. Indeed, Inocentes et al. [35] noted that difficulties in defining the posterior boundary of the nasal cavity and variations in the anatomical landmarks used during segmentation may influence volumetric measurements.

In patients with CLP, maxillary hypoplasia may frequently occur in addition to various nasal deformities, including maxillary sinus anomalies, nasal septal deviation, narrowing of the nasal cavity, and airway obstruction [9,10]. Due to maxillary hypoplasia, orthognathic surgery, particularly Le Fort I osteotomy, may be required as part of the treatment of these patients. Studies conducted in individuals with CLP have reported various changes in nasal morphology following Le Fort I osteotomy [37]. In addition, the nasolacrimal duct may be injured during Le Fort I surgery as a result of osteotomy, drilling, or the placement of osteosynthesis screws [38]. Although the volumetric differences observed in the present study did not remain statistically significant after correction for multiple comparisons, these trends may still provide complementary information regarding individual anatomical variations in the nasomaxillary region. Therefore, three-dimensional assessment of the anatomical characteristics and possible variations of the nasal cavity, maxillary sinus, and nasolacrimal duct in patients with CLP may help identify individual anatomical differences and contribute to treatment planning. However, because the present study was conducted at a single orthodontic referral center, these findings should be interpreted with caution, and their generalizability to the broader CLP population requires confirmation in multicenter studies.

This study has several limitations. First, its retrospective design and the use of CBCT images obtained from a single orthodontic referral center may introduce selection bias and limit the generalizability of the findings to the broader CLP population. Second, significant differences in age and sex distribution were present among the study groups, with the control group being older than both CLP groups. Although age and sex were adjusted for in the statistical analyses, the age difference remains an important limitation because craniofacial structures, including the paranasal sinuses and nasal cavity, may continue to develop during adolescence. Therefore, some of the observed volumetric differences may have been influenced by differences in developmental stage, and residual confounding related to age cannot be completely excluded. This imbalance should be considered when interpreting the between-group comparisons. Third, although all patients with CLP had undergone primary cheiloplasty and palatoplasty during infancy or early childhood, these procedures were not performed by the same surgeon, and detailed information regarding the surgical techniques could not be standardized because of the retrospective design of the study. Therefore, the potential influence of variations in previous surgical reconstruction on maxillary sinus and nasal cavity morphology cannot be completely excluded. Fourth, the relatively small sample size should be considered. As the initial sample size calculation was based on a relatively large expected effect size (Cohen’s f = 0.40), smaller between-group volumetric differences may have remained undetected. Finally, although volumetric measurements were performed using an artificial intelligence-assisted segmentation method, observer reliability ranged from moderate to excellent, with greater variability observed for nasolacrimal duct measurements. The relatively modest agreement observed for some measurements should therefore be considered when interpreting the volumetric findings. Future multicenter prospective studies including larger and more sex-balanced samples are warranted to validate the present findings.

## 5. Conclusions

This study is among the few to simultaneously evaluate the volumes of the maxillary sinus, nasal cavity, and nasolacrimal duct in individuals with unilateral and bilateral cleft lip and palate (CLP) using an artificial intelligence-assisted three-dimensional segmentation approach. Although our results suggest that CLP may exert generalized developmental effects on the nasomaxillary complex, the total-volume comparisons did not remain statistically significant after correction for multiple comparisons, and the reductions that did persist after correction in the side-based analysis were confined to comparisons between the CLP subgroups and the control group; these findings should therefore be confirmed in studies with larger sample sizes.

## Figures and Tables

**Table 1:** Demographic characteristics of the unilateral CLP, bilateral CLP, and control groups.

Group	n	Male n (%)	Female n (%)	Age (Years) Mean ± SD
Unilateral CLP	25	16 (64.00%)	9 (36.00%)	14.77 ± 2.05
Bilateral CLP	20	17 (85.00%)	3 (15.00%)	14.01 ± 1.94
Control	25	8 (32.00%)	17 (68.00%)	18.56 ± 3.21

Data are presented as n (%) or mean ± SD.

**Table 2:** Results of the side-specific mixed-effects model analyses for maxillary sinus, nasal cavity, and nasolacrimal duct volumes.

Volume	Group *p*	Interaction *p*	Gamma *p*	KW(6) *p*	R^2^m/R^2^c
Maxillary Sinus	0.011	0.621	0.641	0.005	0.15/0.76
Nasal Cavity	0.035	0.130	0.043	0.001	0.15/0.73
Nasolacrimal Duct	0.022	0.748	0.672	0.053	0.10/0.78

Primary analysis: age- and sex-adjusted log-linear mixed-effects model. Gamma-GLMM and Kruskal–Wallis (KW) tests were performed as sensitivity analyses. R^2^m/R^2^c = marginal/conditional R^2^.

**Table 3:** Side asymmetry and equivalence analyses of maxillary sinus, nasal cavity, and nasolacrimal duct volumes within the unilateral CLP, bilateral CLP, and control groups.

Volume	Group	A/B Ratio (95%CI)	Mixed *p* (BH)	dz	Result
Maxillary Sinus	Unilateral CLP	1.07 (0.94–1.31)	0.713	0.13	Inconclusive
	Bilateral CLP	1.01 (0.93–1.09)	0.878	0.14	Equivalent
	Control	0.99 (0.93–1.05)	0.878	0.08	Equivalent
Nasal Cavity	Unilateral CLP	1.04 (0.92–1.19)	0.517	0.03	Inconclusive
	Bilateral CLP	0.88 (0.77–1.02)	0.213	−0.34	Inconclusive
	Control	1.04 (0.93–1.16)	0.517	0.12	Inconclusive
Nasolacrimal Duct	Unilateral CLP	0.87 (0.72–1.03)	0.299	−0.20	Inconclusive
	Bilateral CLP	0.95 (0.78–1.15)	0.555	−0.11	Inconclusive
	Control	0.94 (0.81–1.07)	0.555	−0.11	Inconclusive

Ratio = side A/side B. Side A = cleft side in unilateral CLP and right side in bilateral CLP and control groups; Side B = non-cleft side in unilateral CLP and left side in bilateral CLP and control groups. dz = Cohen’s paired effect size. Equivalent = 90% confidence interval within the predefined ±10% equivalence margin (TOST).

## Data Availability

The data presented in this study are available on request from the corresponding author.

## Supplementary Materials

The following supporting information can be downloaded at: https://www.mdpi.com/article/10.3390/jcm15176680/s1, Table S1. Pairwise comparisons of side-specific maxillary sinus, nasal cavity, and nasolacrimal duct volumes among the six subgroups (contrasts of estimated marginal means from the mixed-effects model).

## Abbreviations

AI	Artificial Intelligence
CBCT	Cone-Beam Computed Tomography
DICOM	Digital Imaging and Communications in Medicine
ICC	Intraclass Correlation Coefficient
TOST	Two One-Sided Tests
