# Supplementary material for: Artificial Intelligence-Assisted CBCT Evaluation of Maxillary Sinus, Nasal Cavity, and Nasolacrimal Duct Volumes in Unilateral and Bilateral Cleft Lip and Palate Patients
Source: J Clin Med. 2026 Aug 28;15(17):6680. doi: 10.3390/jcm15176680 (PMC13566317; doi:10.3390/jcm15176680)
Supplement: Supplementary file 1 [file jcm-15-06680-s001.zip › jcm-4473786-supplementary.pdf]

Supplementary Table S1. Pairwise comparisons of side-specific maxillary sinus, nasal cavity, and nasolacrimal duct volumes among the six subgroups (contrasts of estimated marginal means from the mixed-effects model).

Comparison	Ratio (95% CI)	p	BH-adjusted p
Maxillary sinus			
UCLP cleft side vs UCLP non-cleft side	1.07 (0.95-1.21)	0.238	0.357
UCLP cleft side vs BCLP right side	1.14 (0.90-1.46)	0.273	0.372
UCLP cleft side vs BCLP left side	1.16 (0.91-1.47)	0.231	0.357
UCLP cleft side vs Control right side	0.75 (0.58-0.98)	0.036	0.068
UCLP cleft side vs Control left side	0.75 (0.57-0.97)	0.031	0.066
UCLP non-cleft side vs BCLP right side	1.06 (0.84-1.35)	0.608	0.702
UCLP non-cleft side vs BCLP left side	1.08 (0.85-1.37)	0.539	0.673
UCLP non-cleft side vs Control right side	0.70 (0.54-0.91)	0.009	0.023 *
UCLP non-cleft side vs Control left side	0.70 (0.53-0.91)	0.008	0.023 *
BCLP right side vs BCLP left side	1.01 (0.89-1.16)	0.853	0.878
BCLP right side vs Control right side	0.66 (0.49-0.89)	0.007	0.023 *
BCLP right side vs Control left side	0.65 (0.49-0.88)	0.006	0.023 *
BCLP left side vs Control right side	0.65 (0.48-0.88)	0.005	0.023 *
BCLP left side vs Control left side	0.65 (0.48-0.87)	0.004	0.023 *
Control right side vs Control left side	0.99 (0.88-1.12)	0.878	0.878
Nasal cavity			
UCLP cleft side vs UCLP non-cleft side	1.04 (0.92-1.18)	0.502	0.596
UCLP cleft side vs BCLP right side	1.14 (0.90-1.43)	0.281	0.422
UCLP cleft side vs BCLP left side	1.00 (0.79-1.26)	0.993	0.993
UCLP cleft side vs Control right side	0.75 (0.58-0.97)	0.027	0.082
UCLP cleft side vs Control left side	0.78 (0.61-1.01)	0.057	0.122
UCLP non-cleft side vs BCLP right side	1.09 (0.86-1.37)	0.468	0.596
UCLP non-cleft side vs BCLP left side	0.96 (0.76-1.21)	0.730	0.782
UCLP non-cleft side vs Control right side	0.72 (0.56-0.93)	0.012	0.060
UCLP non-cleft side vs Control left side	0.75 (0.58-0.97)	0.027	0.082
BCLP right side vs BCLP left side	0.88 (0.77-1.01)	0.071	0.133
BCLP right side vs Control right side	0.66 (0.50-0.88)	0.005	0.060
BCLP right side vs Control left side	0.69 (0.52-0.92)	0.011	0.060
BCLP left side vs Control right side	0.75 (0.56-1.00)	0.048	0.121
BCLP left side vs Control left side	0.78 (0.59-1.04)	0.088	0.147
Control right side vs Control left side	1.04 (0.92-1.18)	0.517	0.596
Nasolacrimal duct			
UCLP cleft side vs UCLP non-cleft side	0.87 (0.73-1.03)	0.100	0.166
UCLP cleft side vs BCLP right side	1.12 (0.78-1.62)	0.537	0.595
UCLP cleft side vs BCLP left side	1.06 (0.73-1.53)	0.755	0.755
UCLP cleft side vs Control right side	0.62 (0.41-0.92)	0.019	0.049 *
UCLP cleft side vs Control left side	0.58 (0.39-0.86)	0.008	0.038 *
UCLP non-cleft side vs BCLP right side	1.29 (0.90-1.87)	0.167	0.251
UCLP non-cleft side vs BCLP left side	1.22 (0.85-1.76)	0.281	0.383
UCLP non-cleft side vs Control right side	0.71 (0.48-1.06)	0.094	0.166
UCLP non-cleft side vs Control left side	0.67 (0.45-0.99)	0.047	0.101
BCLP right side vs BCLP left side	0.95 (0.78-1.14)	0.555	0.595
BCLP right side vs Control right side	0.55 (0.35-0.86)	0.010	0.038 *
BCLP right side vs Control left side	0.51 (0.33-0.81)	0.005	0.038 *
BCLP left side vs Control right side	0.58 (0.37-0.91)	0.020	0.049 *
BCLP left side vs Control left side	0.54 (0.35-0.86)	0.009	0.038 *
Control right side vs Control left side	0.94 (0.79-1.11)	0.444	0.555

Ratio = back-transformed geometric-mean ratio (first subgroup / second subgroup); values below 1 indicate a smaller volume in the first subgroup. p = unadjusted p-value from contrasts of estimated marginal means; BH-adjusted p = Benjamini-Hochberg-adjusted p-value within each anatomical structure (15 comparisons). * BH-adjusted $p < 0.05$. UCLP = unilateral cleft lip and palate; BCLP = bilateral cleft lip and palate.
